# The Long-Term Public Health Impact of a Community-Based Participatory Research Project for Health Promotion Among Socially Disadvantaged Women—A Case Study Protocol

**DOI:** 10.3389/fpubh.2021.628630

**Published:** 2021-04-12

**Authors:** Karim Abu-Omar, Heiko Ziemainz, Julika Loss, Michael Laxy, Rolf Holle, Ansgar Thiel, Annika Herbert-Maul, Stephanie Linder, Maike Till, Alexandra Sauter

**Affiliations:** ^1^Department of Sport Science and Sport, Friedrich-Alexander-University Erlangen-Nuremberg, Erlangen, Germany; ^2^Department of Epidemiology and Health Monitoring, Robert Koch Institute, Berlin, Germany; ^3^Department of Sport and Health Sciences, Entrepreneurial University Munich, Munich, Germany; ^4^Institute for Medical Informatics, Biometry and Epidemiology, University Hospital, Ludwig-Maximilian-University Munich, Munich, Germany; ^5^Institute of Sports Science, Eberhard-Karls-Universität Tübingen, Tübingen, Germany; ^6^Medical Sociology, Department for Epidemiology and Preventive Medicine, University Regensburg, Regensburg, Germany

**Keywords:** long-term public health impact, physical activity, community-based participatory research, low socioeconomic status, ethnic minorities, vulnerable groups

## Abstract

**Introduction:** Community-based participatory research (CBPR) is considered to be of high potential for health promotion among socially disadvantaged groups. However, the long-term implementation and transfer of these approaches remain challenging, and the public health impact they achieve is difficult to study. This also pertains to the potential health effects and cost-effectiveness of CBPR. This study protocol describes the follow-up case study (NU-BIG) after 15 years of the BIG project (“movement as investment in health”), a project to promote physical activity among socially disadvantaged women. Through a participatory approach, BIG empowers the addressed women to plan and implement low-threshold physical activity offers. Since the project started in 2005, it was transferred to 17 communities in Germany.

**Materials and Analysis:** NU-BIG intends to examine the long-term effects, including economic aspects, of the BIG project on individual and structural levels at all project sites, as well as its long-term implementation and transfer. NU-BIG is a cross-sectional and longitudinal study using a mixed method approach. For the longitudinal section, we re-analyze existing data from former BIG evaluations. For cross-sectional data collection, we use questionnaires and conduct qualitative interviews and focus groups. Women who take part in BIG program offers are part of the research team and will use the photo-voice approach to report on the effects of BIG. The study population consists of about 800 women who participate in BIG project offers and 50 persons involved in the implementation of the BIG project at local sites.

**Discussion:** The expected results from NU-BIG are highly relevant for studying the long-term public health impact of CBPR. In particular, this project intends to answer questions on how the transfer of such projects can succeed and which factors determine if a CBPR project can be sustained at the community level. Eventually, these results can contribute to the further development of participatory approaches to provide effective health promotion among socially disadvantaged groups.

**Conclusion:** Although CBPR is seen of having the potential to reduce health disparities, there is still a lack of research on its long-term effects and public health impact. NU-BIG aims at generating knowledge about the economic effects, reach, efficacy, adoption, implementation, and maintenance of a CBPR project. The expected results could be of high interest for BIG and other CBPR-projects.

## Introduction

Although it is well-proven that physical activity benefits general health (1), many people are insufficiently active. This is particularly true for women of low socioeconomic status (SES) regarding their leisure-time physical activity (2, 3). Precarious living conditions (e.g., living in a deprived area, poverty, or stress) often place a strain on these women's health (4). Given the increased incidence of non-communicable diseases (e.g., cardio–vascular diseases, diabetes mellitus type II) among groups with a low SES, these women can distinctly benefit from the health promoting effects of physical activity on physical and mental health (1, 5). However, there are barriers (e.g., high costs for participation, lack of child care offers) that hinder these women from participating in and benefiting from existing exercise and health promotion programs (6, 7). This necessitates the development of tailored exercise and health promotion programs for this target group.

Reaching women from low-SES background can be considered one of the key challenges in developing such a program. Previous studies have recognized the potential role of participatory research in overcoming this challenge (8, 9). As a research paradigm, community based participatory research (CBPR) engages community members and researchers in the process of taking action to improve community health (10). Thus, through the use of CBPR, it is possible to generate knowledge on effective strategies to reduce health disparities, achieve structural changes at the community level, tailor offers to the interests and needs of the addressed participants, and lower the entry barriers for users (9–11). While the short term effects of participative approaches are reported by a number of projects (8, 9), there is still a lack of national and international studies reporting on the long-term effects of CBPR at behavioral and structural levels.

Such long-term effects of CBPR (e.g., effects on the capacity building among the community, empowerment and the health behavior of addressed users) are of high interest, as these might often occur only years after the implementation of a project (8, 9, 12, 13). Additionally, knowledge on the economic effects and costs of implementation of CBPR are still scarce (9). To investigate the potential broader (societal) influence of CBPR, there is a substantial need for studies that follow up on CBPR projects. Furthermore, long-term studies of CBPR can shed light on how such highly context-specific projects can be transferred and scaled-up (14–16).

The CBPR project BIG (“Bewegung als Investition in Gesundheit” = Movement as Investment in Health) has the potential to demonstrate the public health impact and scale-up of participatory approaches. This study protocol describes the follow-up study of BIG: NU-BIG (“Nachuntersuchung des BIG-Projekts”).

## The Big Project

The BIG-project was started in 2005 by the Department of Sport Science and Sport of Friedrich-Alexander-Universität Erlangen-Nuremberg, Germany (FAU). The BIG project intends to develop and implement physical activity promotion programs for women in difficult life situations, for example those who have a low household income, have a migration background, are unemployed, rely on welfare aid, or are single mothers. Using CBPR, the BIG project aims at promoting physical activity among these women by engaging them in the process of planning and implementing exercise programs with very low entry barriers. Additionally, by using CBPR, BIG intends to empower these women to take control of their own health (17, 18).

The project's method for initiating women's participation is referred to as a “cooperative planning approach” (12). This method intends to equally involve members of the target groups in the project-that is, women, researchers, and community-level stakeholders of policy and practice (e.g., mayor of the community/city; representatives of sports clubs; trainers). Over the course of various planning sessions, women work with researchers and stakeholders to define goals and plan exercise programs (18). As all members of the cooperative planning provide specific resources (e.g., funds, access to sport facilities, contact information for the addressed women), it is possible to implement these programs on the community level. It is intended that these planning sessions will empower the women in the target group, expand the social networks of everybody involved, and make stakeholders more aware of the needs of these women. Furthermore, women in the target group can gain expert knowledge on the organizational and political processes related to planning health promotion programs at the community level, thus increasing their self-efficacy (19). Additionally, cooperative planning results in a support network of stakeholders to ensure long-term implementation and sustainability at the community level. This includes assigning a person in the city administration the role of coordinating project activities and serving as a point of contact for all partners and interested persons (20).

BIG program offers, resulting from the cooperative planning, range from exercise classes with low entry barriers (e.g., free of charge, availability of childcare, close to the places where the women live), such as dancing, swimming, or fitness classes, to the creation of new physical activity opportunities, such as women-only indoor pool hours. BIG program offers are promoted by everyone involved, especially importantly including women from the target group, who inform and engage with other women in their daily life-for example at kindergarten or places of worship-thereby drawing their own social networks to these classes.

Since 2005, the BIG project, which originated in Erlangen (Germany), has been transferred to 16 additional sites in Germany. In the first years, the German federal ministry of education and research funded the BIG project; later on, a variety of different funders financed BIG and provided seed funding for new BIG sites (20). To date, BIG has been successfully sustained in seven communities, and in total around 800 women take part in BIG program offers. Nine other communities were able to implement BIG (for an average of about 4 years), but the project could not be sustained. One additional community is currently preparing to implement BIG (20).

## Aims and Objectives

NU-BIG, the follow-up of the BIG project is intended as a comprehensive cross-sectional and longitudinal evaluation of all BIG sites. It aims to gain insight into the long-term effects of the BIG project at the individual (women and stakeholders taking part in the project) and structural levels (changes in how the city administration plans health promotion offers), as well as on its economic effects, sustainable implementation, and transfer to other sites. As a CBPR project, women in difficult life situations are involved in all phases of this study.

As a theoretical framework for the long-term evaluation of BIG, Glasgow et al. (21)'s RE-AIM model is applied. RE-AIM was chosen because it provides a comprehensive framework for evaluating the impact of public health interventions. In particular, it allows researchers to focus on whom an intervention reached and how an intervention is being adopted by other communities, two crucial components of BIG. According to this model (21), the public health impact of an intervention depends on five dimensions: reach, efficacy, adoption, implementation, and maintenance.

Our research questions can be located accordingly in the RE-AIM framework:

**Table:** 

Reach:	Has BIG been reaching women in difficult life situations?
Efficacy:	What effects does the participation of women in BIG exercise classes or the planning process of BIG have on their health and behavior (for example physical activity levels), social networks, and empowerment? Which economic determinants and effects are associated with the long-term implementation of BIG?
Adoption:	What lessons can be learned from the implementation of BIG within local communities and the behavioral and structural impacts of BIG for a successful transfer and roll out to additional communities?
Implementation:	Which determinants are responsible for a successful and sustainable implementation of BIG at different sites? What costs are associated with a sustainable implementation of BIG?
Maintenance:	What behavioral and structural effect does BIG have on the local community? What factors can explain why some communities are able to sustain the BIG project longer than others?

## Methods and Analysis

### Study Design

**Figure 1:**
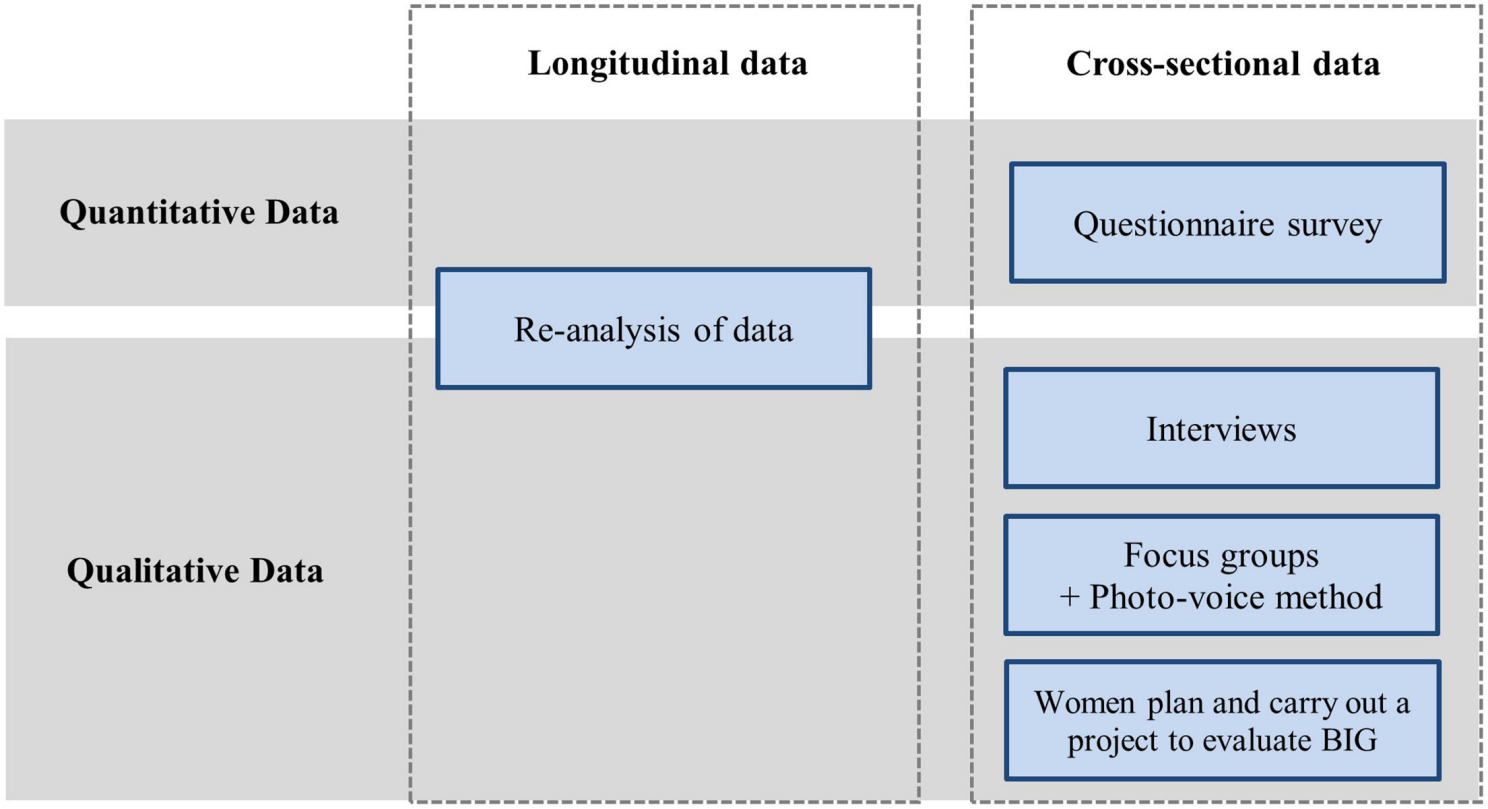
Study design of NU-BIG.

In the context of NU-BIG, cross-sectional and longitudinal data collection using mixed methods of qualitative, quantitative, and participative methodologies will take place (Figure 1).

#### Longitudinal Data

The original data for the longitudinal evaluation was obtained from previously conducted surveys, administered during the course of various projects to transfer BIG. Researchers based out of FAU have evaluated different aspects of BIG at various sites over the years (e.g., the ability to reach the targeted women, effects of BIG on the women's physical activity), but have never captured comprehensive data over all BIG-sites. Methodologically, medical tests, standardized questionnaires, qualitative interviews and focus groups, and self-assessment questionnaires were administered. Data was gathered from the women belonging to the target group, project coordinators, and stakeholders. Survey parameters were, among others, the effectiveness of BIG exercise classes on health, health behavior, risk factors, and social networks, as well as costs related to the project, ability to reach the target population, and indicators for sustainable implementation at project sites (22, 23).

As a first step, NU-BIG will re-analyze this data:

a) to identify people who played a key role in the implementation of BIG at the different sites,
b) to detect previously-used measurement tools in order to develop the questionnaire and interview guides for the cross-sectional study and
c) to gather data regarding the research questions.

#### Cross-Sectional Data

##### Settings and Population

Cross-sectional data will be collected from the seven “active” communities (= BIG exercise classes are currently running) as well as the ten “inactive” sites (= BIG classes are no longer offered). Within the active communities, all women currently taking part in BIG classes, as well as some individuals who participated in the past, will be interviewed by questionnaires. Currently, ~800 women are actively participating in BIG offers. Women who participated in BIG-classes in the past, at the inactive communities will be interviewed in focus groups. These women will be contacted by former project-coordinators and exercise constructors from these sites. Also, stakeholders responsible for the coordination or implementation of BIG (i.e., stakeholders, supervisors of BIG project coordinators, exercise instructors) at all current and former project sites will be interviewed. This group will comprise a total of ~50 people.

### Study Method

Data for NU-BIG will be collected using (a) questionnaires, (b) interviews, and (c) focus group interviews combined with a Photovoice approach (see Table 1). Additionally, the questionnaire will be used to collect data for the health economic analysis.

a) It is planned to conduct a survey among all 800 participants of the BIG classes. Based on the experiences of previous questionnaire surveys, we expect a 50–60% response rate, which corresponds to about 420 completed questionnaires. Participants will be asked to take part in the survey during BIG classes. Questionnaires will be translated into different languages.
b) Semi-standardized interviews are planned to be held with ~47 people. Interviewees will be participants of BIG activities, BIG coordinators, supervisors of BIG coordinators, as well as peers of the active and inactive BIG sites. Additionally, it is planned to interview the researchers that worked on the implementation and evaluation of the project in the past.
c) Focus group interviews (*n* = 5), and one focus group interview in combination with Photovoice, will also be utilized to collect data from the women (24). A group of women (minimum *n* = 6) will be asked to take photos representing changes in their lives due to their participation in BIG. For example, this may include pictures of other people that became part of their social network because of BIG. These pictures will be the basis for discussion during the focus group and may help women reflect on changes they experienced through BIG.

**Table 1:** Overview of study methods, participants, and survey parameters.

	**Quantitative data collection**	**Qualitative data collection**	**Cluster focus group**	**Photo-voice approach**
Participants of active locations (approx. 7)	*N* = approx. 800 Content: - Socio-economic status - Wellbeing - Empowerment - Class bonding - Structure of social network - Satisfaction	*N* = approx. 7 Content: - Long-term effects of classes - Structure of social network - Low threshold of offers - Satisfaction with offers - Evaluation product of women	*N* = approx. 2–3 Content: - Effects of classes - Structure of social network - Satisfaction with offers - Estimation of necessity of BIG	*N* = approx. 6 Content: - Long-term effects of classes
Former participants of active locations		*N* = approx. 5- effects of classes - Reasons for quitting classes		
Former participants of inactive locations (ca. 10)		*N* = approx. 5 Content: - Low threshold of offers, - Satisfaction with offers - Valuation of BIG	*N* = approx. 1–2 Content: - Effects of classes - Structure of social network - Satisfaction with offers - Estimation of necessity of BIG	
Project coordination Head of office/peers /Trainers of active and inactive locations		*N* = approx. 30 Content: - Local structural development - Modification of BIG - Mobilization of resources - Assurance of financing - Identification of facilitating factors for sustainability and transferability - Barriers regarding implementation of BIG		
Number of Participants	Approx. 800	Approx. 47	Approx. 4	Approx. 6

To increase the response rate for the survey and the qualitative interviews, questionnaires will use easy language and, if necessary, be translated into different languages relevant to the target-group. Interpreters will be used during interviews, where needed, to minimize language barriers between interviewer and interviewees. This is considered helpful in minimizing reservations to the study (25). Current coordinators and peers will recruit potential participants for the data collection.

### Study Parameters

Table 1 presents an overview of study participants, methodologies, and parameters within the RE-AIM framework:

To evaluate the *Reach* of BIG, we will collect data on the socio-economic status of women as well as their satisfaction with BIG exercise classes. Women who no longer take part in BIG classes will be asked to state their personal reasons for discontinuing.

*Efficacy* of the project will be investigated by asking about the participants' well-being, empowerment, social network, and physical activity behavior, as well as by exploring any long-term-effects due to BIG. Additionally, NU-BIG will measure what health and economic effects are connected to BIG as well as which structures were established within these settings.

Interviews with local coordinators and stakeholders of BIG will enable the evaluation of the *Adoption* of the project within each setting. By doing so, we might be able to identify which factors are relevant for a positive decision on locally implementing the BIG project.

NU-BIG will evaluate *Implementation* by measuring the factors, which were responsible for a successful realization of the project, including what modifications were made to the BIG approach, and what costs are associated with its sustainable implementation. Additionally, it will evaluate what resources were available to implement BIG in the different communities as well as who funded the project on the local level.

To learn about the project's *Maintenance*, data will be collected on the women's long-term participation in exercise programs, the measures communities took and potential structural changes in communities to maintain BIG.

### Participation

Based on the participative nature of BIG, the evaluation project NU-BIG will also involve the target group extensively at all steps. To ensure this, one woman who is taking part in BIG exercise classes, from each community where classes are offered, will be hired as an expert to join evaluation efforts of NU-BIG. A total of seven women will serve as part of the research team and represent the target-group's perspective during both method development and the planning phase of data collection. This is done to ensure the interests of the target group are met in the evaluation and that all evaluation instruments (e.g., questionnaires) are acceptable and easy to understand. These women are of vital importance to the project's success during data collection, as they serve as the key link between researchers and women taking part in BIG classes. Women experts are compensated for their efforts with an allowance of €1,500 per year, per woman, for a term of 3 years.

Importantly, these women receive a budget of €5,000 to create an evaluation project of their choice, which showcases potential benefits of BIG for them. The aim of the project is to show the benefits and long-term effects of BIG through the lens of the target group. For example, women might choose to create a documentary or movie, write a book, or organize an art exhibition about BIG.

### Analysis

The quantitative analysis will use descriptive and multivariate analyses to assess the effects of the exercise classes on the women's health parameters. Additionally, variance analyses will be performed to measure the influence of the following variables on women's health:

- Project sites (to identify regional differences)
- Duration of project implementation
- Participants at the BIG sites

Regression analysis will be used to investigate factors predicting the long-term exercise adherence of the women.

Regarding qualitative data, interviews and focus groups will be recorded and transcribed. Evaluation will be based on the grounded theory approach (26)—that is, transcripts are read repeatedly and the content is sequentially analyzed; identified concepts and context are combined into sub-categories and later combined into key categories. Pictures for the focus group will be taken according to Wang (27)'s photo-voice approach, and will then be discussed and evaluated accordingly.

## Discussion

Until now, research on the long-term effects, costs, and transferability of CBPR with socially disadvantaged population groups has been scarce (9). NU-BIG, the follow-up study of the BIG project described herein, offers an opportunity to address this research gap. NU-BIG has the potential to produce insights that are not only of interest for the BIG project but also for any future CBPR project intending to scale and to reflect upon the public health impact it is generating (9, 15).

NU-BIG will produce research outcomes on (a) how to reach people in difficult life-situations, (b) the long-term effects of participatory approaches on behavioral and structural levels, and (c) the determinants of a sustainable implementation and transfer to other settings. Thus, the results of NU-BIG allow conclusions to be drawn about the public health impacts of BIG (21).

Project outcomes can be utilized to modify the methods applied in BIG to include women in the planning of exercise classes. Furthermore, it will be used for the development of a scaling-concept to increase the public health impact of BIG, and to point out options for a potential transfer to other settings (e.g., worksites) or different target groups (e.g., men in difficult life situations) (16).

A strength in the evaluation of 15 years of project activities lies in having focused not only on direct health outcomes related to increased physical activity, but also on outcomes generated by engaging women in the planning of classes and giving stakeholders the opportunity to learn more about the needs of these women (11). This knowledge is needed to understand the mechanisms and potential effects of participatory approaches, particularly among socially disadvantaged groups (28).

Furthermore, comparing the evidence on the potential long-term effects on health, and the costs of program implementation and transfer will allow the economic characteristics of the project to be assessed. These insights will be beneficial for potential funding agencies considering supporting the transfer of CBPR projects (9, 28).

NU-BIG also provides insights on the determinants for the successful long-term implementation and transferability of CBPR, which are important in increasing the public health impact of such projects. The expected outcomes are highly compatible with current scientific discourse on topics, such as scaling-up, and other participative approaches (e.g., citizen science, community-based participatory research).

## Limitation and Strengths

Several limitations of this study should be mentioned:

(1) Data collection from communities where BIG has ceased to exist may be limited. Recruiting survey participants at these locations will be difficult. Former stakeholders and/or former participants of exercise classes may refuse to take part in the survey or interviews.
(2) Language barriers will persist. It must be expected that potential participants will refuse to participate in the study due to language barriers or reservations about sharing personal data. To keep these barriers to a minimum, attention has been paid to the comprehensibility of the survey instruments and they have been translated into various relevant languages. This may not be sufficient, however, as women from many different countries of origin (with many different native languages) participate in BIG courses.
(3) Due to the retrospective nature of data collection, a recall bias cannot be ruled out. It may be the case that respondents no longer remember relevant information, and thus do not mention it. In some communities, the BIG project was conducted years ago.
(4) RE-AIM as an evaluation framework fits CBPR only to a limited extent. RE-AIM is mainly suitable for standardized interventions. It has its limits when applied to participatory interventions, as such, interventions have a much broader understanding of what constitutes “efficacy.” On the other hand, RE-AIM is strong in quantifying the reach and adoption of interventions and thus their public health impact. The present project is more interested in investigating why (or why not) sustainability could be achieved at different locations.

## Conclusion

CBPR is recognized as a potent research paradigm to reduce health disparities. Yet, there is a lack of research on the long-term health effects, system changes, sustainability, transfer, and cost- effectiveness of CBPR and thus its public health impact. Using the BIG-Project (Movement as investment in health) as a case study, this study protocol describes the follow up (NU-BIG) after 15 years of project implementation at various sites. NU-BIG uses a cross-sectional and longitudinal study design with mixed methods and participatory approaches. The aim of NU-BIG is to generate knowledge about the economic effects, reach, efficacy, adoption, implementation, and maintenance of BIG, and thus about the project's public health impact. The results are expected to identify factors, which contribute to public health impact and a scaling-up concept to transfer the project to new sites or target groups. NU-BIG is therefore expected to provide insights that could be highly interesting for other CBPR-projects.

## Ethics Statement

The process to get ethical approval for the study from the ethics committee at Friedrich-Alexander University Erlangen-Nuremberg is in progress. The ethics committee of FAU will be able to issue ethical approval once the measurement instruments of the study have been decided upon. Due to the participatory approach of this research project, women of the target group are involved in decisions on endpoints and how to measure those. Thus, measurement instruments have not yet been decided upon. Once instruments will have been developed with the consensus of women, these will be submitted to the local ethics committee for approval. Results will be published in relevant journals on national and international levels and presented at national and international conferences. Additionally, there will be a closing event including all researchers and participants of the study where empirical outcomes will be presented. The outcomes will be accessible to the public through a project's website and a newsletter. The BIG Manual, which is currently used as a guide for new locations, will be revised with new information for future interested communities.

## Author Contributions

KA-O and HZ initiated, conceived the study, and participated in writing the manuscript. JL, ML, RH, AT, and AS contributed to study conception and critically revised the manuscript. AH-M drafted the manuscript. MT and SL contributed to study conception and revised the manuscript. All authors read and approved the final manuscript.

## Conflict of Interest

The authors declare that the research was conducted in the absence of any commercial or financial relationships that could be construed as a potential conflict of interest.

## Abbreviations

BIG: *Bewegung als Investition in Gesundheit* (movement as investment in health)
NU-BIG: *Nachuntersuchung von BIG* (follow-up study of BIG)
CBPR: community-based participatory research
RE-AIM: reach, efficacy, adoption, implementation, maintenance.
